# Oral Microbiota Associated With Parkinson'S Disease: A Systematic Review

**DOI:** 10.1111/scd.70272

**Published:** 2026-10-09

**Authors:** Myllenne dos Santos Abreu, Rayane Portela Lima de Morais, Tácio Fragoso Pereira, Pedro Henrique da Hora Sales, Jair Carneiro Leão, Alessandra de Albuquerque Tavares Carvalho

**Affiliations:** ^1^ Department of Dentistry Federal University of Pernambuco Recife Pernambuco Brazil; ^2^ Department of Dentistry Federal University of Alagoas Maceió Alagoas Brazil

**Keywords:** biomarkers, chronic disease, dysbiosis, microbiota, neurobehavioral manifestations, oral cavity, Parkinson's disease

## Abstract

**Aims:**

To evaluate the available evidence on the association between oral microbiota composition and Parkinson's disease (PD), including its relationship with disease severity and clinical features.

**Methods and Results:**

This systematic review was registered in PROSPERO (CRD42024560059) and conducted according to the PRISMA 2020 statement. Searches were performed in PubMed, Embase, Web of Science, LILACS, Scopus, and SciELO. Observational studies evaluating the oral microbiota of adults with PD were included. Eleven studies, predominantly case‐control, were analyzed, with 16S rRNA sequencing used in 90.9% of them. The genera most frequently associated with PD were *Lactobacillus* (45.45%), *Prevotella* (36.7%), and *Streptococcus* (36.7%). Most studies reported differences in beta diversity, indicating a distinct oral microbial profile in patients with PD. Higher *Lactobacillus* abundance and lower *Neisseria* abundance were associated with greater disease severity and poorer cognitive performance in some studies. According to the GRADE approach, the certainty of the evidence was very low.

**Conclusion:**

The available evidence suggests that PD is associated with alterations in oral microbiota composition. However, because the evidence is derived predominantly from observational studies, causal relationships cannot be established. Longitudinal studies using standardized microbiome methodologies are needed to validate these microbial signatures as candidate biomarkers.

## 1 Introduction

Parkinson's disease (PD) is a progressive neurodegenerative disorder characterized by the early degeneration of dopaminergic neurons in the substantia nigra [1, 2]. Although its cause is still unknown, the main histopathological alteration observed is the accumulation of α‐synuclein, which leads to neural destruction and a reduction in dopamine. These alterations result in movement disorders, manifested through the classic motor symptoms of PD, including tremor, rigidity, bradykinesia or akinesia, postural instability, and slowness of movement [3, 4, 5].

As the second most prevalent neurodegenerative disease in the elderly [6], PD is also associated with stomatognathic diseases such as caries, periodontitis, and temporomandibular disorders [5], along with specific oral manifestations, including xerostomia, sialorrhea, and swallowing difficulties [7]. The clinical picture of PD extends to a wide range of motor and non‐motor manifestations, including psychiatric, genitourinary, gastrointestinal and cardiovascular problems [4].

The associations between PD and the human microbiome at different mucosal interfaces, such as the nose, mouth and gut, have generated increasing interest in recent years [8]. Although much of the literature focuses on changes in the gut microbiota, studies suggest that the oral cavity also plays a crucial role in the relationship between microbiota and neurological conditions [9, 10, 11, 12, 13, 14, 15, 16, 17, 18, 19].

Furthermore, as it is the main gateway for ingested materials and has a rich vascularization [20], the oral cavity has great potential for introducing pathogens capable of affecting the central nervous system [21]. In this context, oral dysbiosis stands out as a relevant factor not only in the emergence of oral diseases, but also in the development of conditions that affect systemic health [22].

As we age, the oral microbiome undergoes changes that increase the abundance of pathogens, making individuals more susceptible to oral diseases [23, 24]. Tooth loss, for example, significantly impacts the oral cavity by eliminating key habitats that support microbial diversity, such as tooth surfaces and subgingival areas [25, 26]. In addition, the prolonged use of dentures in old age influences the quantity and composition of microbial species present in the oral cavity [24].

Therefore, it is highly suggestive that oral pathogens may be associated with Parkinson's disease through specific oral conditions, such as periodontitis. There is evidence suggesting an association between PD and periodontal disease, with *Porphyromonas gingivalis* being one of the microorganisms most frequently implicated in inflammatory mechanisms that may contribute to dopaminergic neurodegeneration in PD [26, 27, 28].

It is therefore relevant to investigate whether specific microorganisms present in the oral cavity are associated with Parkinson's disease and its clinical manifestations. Previous studies have reported alterations in salivary secretion, saliva composition, and oral microbial composition in patients with PD [29]. Because saliva represents a promising source of biomarkers for chronic diseases [30], the characterization of the oral microbiota may contribute to a better understanding of its association with Parkinson's disease and its clinical features.

Considering the growing evidence pointing to interactions between the oral environment and neurological conditions, this systematic review aimed to evaluate the available evidence on the association between oral microbiota composition and Parkinson's disease, including its relationship with disease severity and clinical features.

## 2 Materials and Methods

This systematic review was registered in PROSPERO (CRD42024560059) and conducted in accordance with the Preferred Reporting Items for Systematic Reviews and Meta‐Analyses (PRISMA 2020) statement [31].

The eligibility criteria were established according to the PECOS framework. The review was designed to answer the following research question: *What is the available evidence on the association between oral microbiota composition and Parkinson's disease and its relationship with disease severity and clinical features?* The population (P) comprised adults diagnosed with Parkinson's disease; the exposure (E) was oral microbiota composition or oral dysbiosis; the comparator (C) included healthy controls or comparisons across different levels of disease severity, when available; the outcomes (O) were oral microbial composition, alpha and beta diversity, microbial taxa associated with Parkinson's disease, and clinical features; and the study design (S) included case‐control, cross‐sectional, and cohort studies.

Eligible studies were observational (case‐control, cross‐sectional, or cohort) investigations evaluating the oral microbiota of adults diagnosed with PD. Studies assessing oral microbial composition, whether under dysbiotic or normobiotic conditions, and reporting associations with PD were considered for inclusion. Only studies published within the previous ten years were included to reflect advances in high‐throughput sequencing technologies and the evolving understanding of oral dysbiosis in PD.

Case reports, case series, animal studies, preclinical studies, studies involving pediatric populations, and articles with insufficient methodological information were excluded.

A meta‐analysis was not feasible because of substantial methodological heterogeneity among the included studies, including differences in sampling sites (saliva, tongue, subgingival plaque, dental plaque), sequencing methods (different 16S hypervariable regions and platforms), taxonomic levels reported, microbial outcome measures, and inconsistent reporting of effect estimates. Therefore, a narrative synthesis was considered the most appropriate approach.

### 2.1 Search Strategy

A comprehensive search strategy was developed to identify studies investigating the association between the oral microbiota and Parkinson's disease (PD). Searches were conducted in PubMed, Embase, Web of Science, LILACS, Scopus, and SciELO between April and June 2025. The search strategy was adapted for each database and combined controlled vocabulary (MeSH and Emtree terms, when applicable) with free‐text terms related to Parkinson's disease and the oral microbiota using the Boolean operators AND OR. The complete search strategies for all databases are presented in Table 1 to ensure reproducibility.

**TABLE 1:** Complete search strategies used for each electronic database.

Database	Search strategy
PubMed	((“Parkinson Disease”[Mesh] OR Parkinson[Title/Abstract]) AND (“oral microbiota”[Title/Abstract] OR “oral microbiome”[Title/Abstract] OR (“oral”[Title/Abstract] AND (microbiota[Title/Abstract] OR microbiome[Title/Abstract])) OR (“saliva”[Title/Abstract] AND (microbiota[Title/Abstract] OR microbiome[Title/Abstract])) OR (“gingiva”[Title/Abstract] AND (microbiota[Title/Abstract] OR microbiome[Title/Abstract])) OR (“dental plaque”[Title/Abstract] AND (microbiota[Title/Abstract] OR microbiome[Title/Abstract])) OR dysbiosis[Title/Abstract])) AND (Parkinson Disease[MeSH Terms])
Embase	(‘Parkinson disease’)/br AND (‘mouth flora’) OR ((‘oral microbiome’):ab, ti) AND ((‘microflora’):ab, ti) OR ((‘microbial interaction’):ab, ti) AND ((‘dysbiosis’):ab, ti)
Web of science	Parkinson Disease (Title) or Parkinson (Topic) and oral microbiota (Topic) or oral microbiome (Topic) or dysbiosis (Topic) and oral (Abstract)
Scopus	(TITLE‐ABS‐KEY (Parkinson disease) OR TITLE‐ABS‐KEY (Parkinson s disease) OR ALL (Parkinson disease) AND TITLE‐ABS‐KEY (microbiota) OR TITLE‐ABS‐KEY (microbiome) AND TITLE‐ABS‐KEY (oral) OR TITLE‐ABS‐KEY (oral microbiota) AND TITLE‐ABS‐KEY (dysbiosis))
LILACS	(Parkinson disease) OR (Parkinson) AND (oral microbiota) OR (microbiota) OR (microbiome) AND (oral) AND (dysbiosis)
SciELO	(Parkinson disease) OR (Parkinson) AND (oral microbiota) OR (microbiota) OR (microbiome) AND (oral) AND (dysbiosis)

### 2.2 Screening, Selection and Extraction of Studies

The study selection was conducted in two stages. Initially, two reviewers independently screened the titles and abstracts of all retrieved records according to the predefined inclusion and exclusion criteria. Subsequently, the full texts of potentially eligible studies were independently assessed by the same reviewers. Any disagreements during the selection process were resolved through discussion and consensus. No automation tools were used in the study selection process.

Data extraction was performed independently by two reviewers using a standardized data extraction form. The following information was collected from each included study: author(s), year of publication, country, study design, sample characteristics, oral microbiota associated with PD, sequencing method used, and alpha and beta diversity. Discrepancies in data extraction were resolved by consensus, with re‐evaluation of the original articles when necessary.

The outcomes of interest included oral microbial composition, alpha and beta diversity, microbial taxa associated with Parkinson's disease, and reported associations with disease severity and clinical features.

No data transformations or conversions were required, as results were extracted and presented as reported in the original studies.

Study characteristics and main findings were summarized in tables, and graphical representations were used to illustrate the frequency of reported microorganisms and diversity findings.

### 2.3 Risk of Bias Assessment and Quality of Scientific Evidence

The risk of bias was assessed based on the criteria of the Newcastle‐Ottawa Scale [32], which is divided into three dimensions: selection, comparability and evaluation of the outcome.

In the Newcastle‐Ottawa Scale, the maximum quality score is 9 points. Studies with a score ≥ 7 were considered to have a low risk of bias, scores of 5 to 6 indicated a moderate risk of bias and scores <5 indicated a high risk of bias. The analysis of the included studies was carried out independently by two reviewers (M.S.A. and T.F.P.). Disagreements were resolved by a third reviewer (R.P.L.M.). The quality of evidence was assessed using the Grading of Recommendations, Assessment, Development, and Evaluation (GRADE).

## 3 Results

The database search strategy resulted in a total of 1024 references, 686 of which came from PubMed, 179 from Embase, 126 from Web of Science, 24 from Scopus and 9 from LILACS. After removing 271 duplicate records, 753 titles and abstracts were submitted for initial screening. Of these, 14 articles were selected for full reading. In addition, 1 article was identified from the gray literature via Google Scholar. After a thorough analysis, 11 studies met the inclusion criteria and were incorporated into the final sample of this systematic review.

The entire study selection process is shown in Figure 1.

**FIGURE 1:**
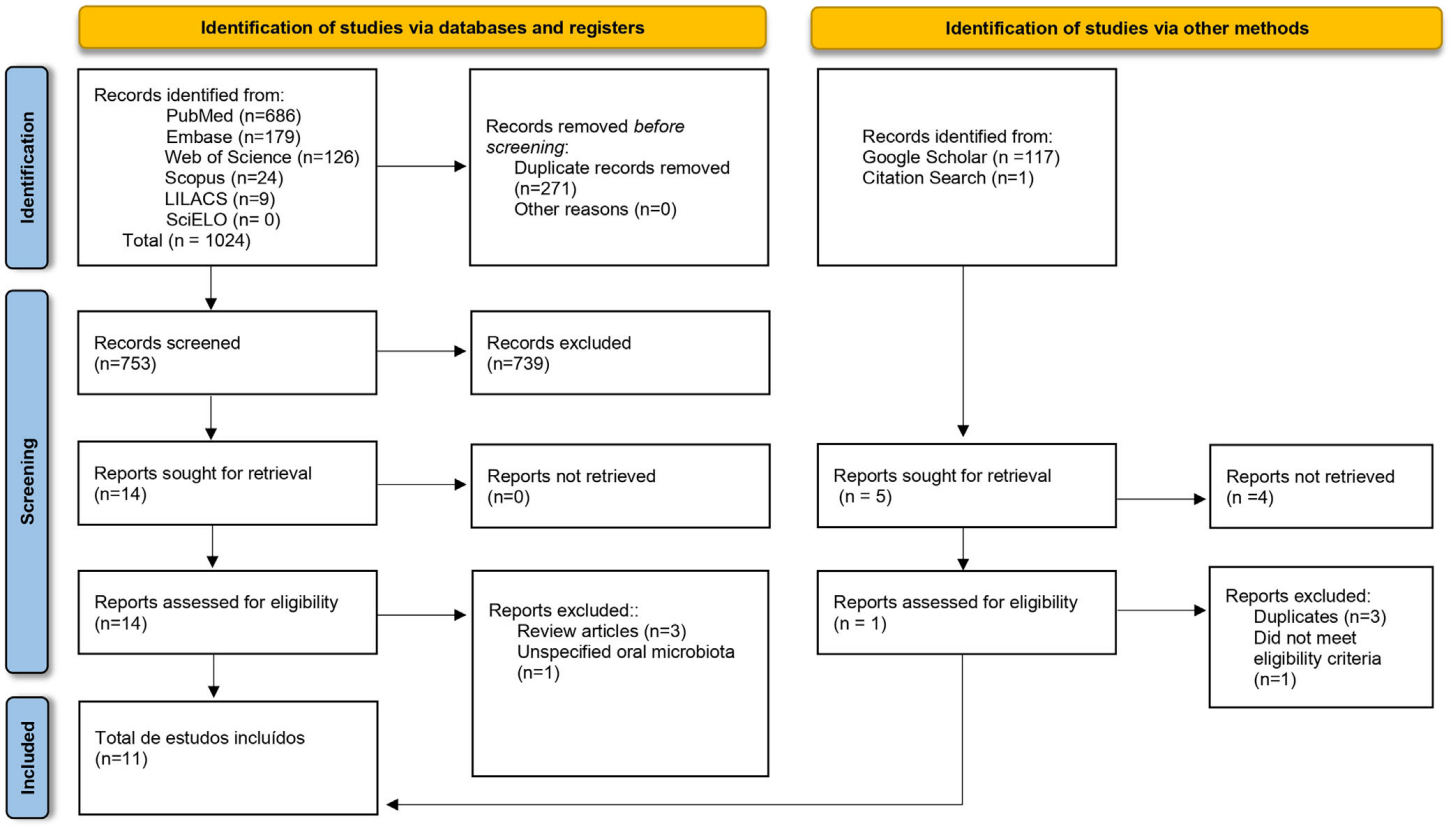
Flow diagram.

### 3.1 Characteristics of Included Studies

A total of 1160 individuals were analyzed in the 11 studies, of which 800 referred to 8 case‐control studies [9, 10, 11, 12, 13, 14, 17, 19] and 360 of 3 cross‐sectional studies [15, 16, 18]. Most of the studies included in this review were conducted in Asia (*n* = 6), with Turkey standing out with three studies [16, 17, 18]. The United States followed with two studies [9, 11], as did China [13, 19]. Other countries represented include Switzerland, South Korea, Finland and Poland, each contributing one publication [10, 12, 14, 15]. More information is described in Table 2.

**TABLE 2:** Characteristics of the included studies.

Study	Design	Country	Sample	Oral microbiota associated with PD
Pereira et al. [14]	Case‐control	Finland	72 cases/76 controls	Increased in PD: *Prevotella, Prevotellaceae, Solobacterium, Coriobacteriaceae, Veillonellaceae, Lactobacillaceae*, and *Veillonella*. Decreased: *Capnocytophaga, Rothia, Kingella, Leptotrichia, Actinomyces, Leptotrichiaceae, Haemophilus, Neisseria, Gemella, Corynebacterium, Granulicatella, Pasteurellaceae, Neisseriaceae, Micrococcaceae, Carnobacteriaceae*, and *Corynebacteriaceae*.
Mihaila et al. [9]	Case‐control	USA	48 cases/36 controls	Increased levels of *Lactobacillus (L. fermentum), Bifidobacterium (B. longum)*, and *Parascardovia denticolens* in early‐stage PD. A decrease in *Streptococcus phage PhiSpn 200* and increased levels of yeasts: *Candida albicans, C. dubliniensis*, and *Saccharomyces cerevisiae*.
Rozas, Tribble & Jeter [11]	Case‐control	USA	30 cases/30 controls	Soft tissues showed increased abundance of *Lactobacillus (L. fermentum, L. acidophilus), Streptococcus (S. pneumoniae, S. vestibularis), Tannerella forsythia*, and *Prevotella intermedia*. In hard tissues: *Fusobacterium nucleatum, Prevotella nigrescens, Campylobacter rectus*, and *Neisseria*.
Fleury et al. [10]	Case‐control	Switzerland	20 cases/20 controls	PD patients had higher abundance of *Streptococcus mutans, Kingella oralis, Actinomyces, Veillonella, Scardovia, Lactobacillaceae, Negativicutes*, and *Firmicutes*. Lower abundance of *Treponema KE332528_s* and *Lachnospiraceae AM420052_s*.
Jo et al. [12]	Case‐control	South Korea	91 cases/85 controls	*Lautropia, Abiotrophia*, and *Oscillibacter* were less frequent in PD; *Lactobacillus, Capnocytophaga*, and *Megasphaera* were more prevalent. In severe PD, increases in *Haemophilus* and *Lactobacillus*, and reduction in *Lautropia*.
Li et al. [13]	Case‐control	China	91 cases/91 controls	Higher abundance in PD patients: *Firmicutes, Bacilli, Lactobacillales, Streptococcaceae, Streptococcus, S. mitis*, and *Prevotellaceae*. Greater presence of *Neisseria subflava* was associated with milder motor and non‐motor symptoms.
Yay et al. [17]	Case‐control	Turkey	20 P/20 P+PD/20 controls	PD patients showed greater prevalence of periodontal pathogens, such as *P. gingivalis, Tannerella forsythia, Prevotella spp., Eubacterium spp., Selenomonas spp., Campylobacter, Fusobacterium spp*., and *Treponema spp*.
Li et al. [19]	Case‐control	China	35 cases/15 controls	Mild PD patients had higher levels of *Prevotella, Lactobacillus, Megasphaera, Atopobium*, and *Howardella*, negatively correlated with cognitive function.
Zapata et al. [15]	Cross‐sectional	Poland	59 cases/108 controls	PD patients had higher levels of *Prevotella, Streptobacillus, Megasphaera, Lactobacillus, P. histicola, P. melaninogenica*, and *P, gingivalis*. Healthy controls had more *Haemophilus*, *TM7*, and *Veillonella*.
Arikan et al. [16]	Cross‐sectional	Turkey	142	Patients with cognitive decline had reduced *Neisseria, Neisseriaceae, Alloprevotella, TM7x*, and *Fusobacterium*, and increased *Ligilactobacillus, Lactobacillus, Rothia, Corynebacterium, Gemella, Streptococcus*, and *Veillonella*.
Yay et al. [18]	Cross‐sectional	Turkey	18 P/16 P+PD/17 controls	Most abundant species in PD: *Mycoplasma faucium, Tannerella forsythia, Parvimonas micra*, and *TM7* subspecies. P+PD group showed increased *Prevotella pallens* and *P. melaninogenica*.

The overall scores of the studies ranged from 5 to 9 stars, indicating a methodological quality that varies between moderate and high. The study by Rozas, Tribble and Jeter [11] stood out for its consistency, achieving the maximum score, while the study by Zapata et al. [15], Arikan et al. [16] and Yay et al. [18] achieved 5 points, presenting a higher risk of bias. Despite some variations in the individual scores, there was relative homogeneity in the quality of the studies, with the majority obtaining high scores according to the Newcastle‐Ottawa Scale. According to the assessment based on GRADE criteria, the certainty of evidence related to the Association between dysbiosis/periodontal pathogens and Parkinson`s disease was very low. Further explanations regarding the evidence assessment are available in Table 3.

**TABLE 3:** GRADE assessment of the certainty of evidence for the included outcomes.

Certainty assessment Association between dysbiosis/periodontal pathogens and Parkinson's disease								
Studies	Study design	Risk of bias	Inconsistency	Indirect evidence	Imprecision	Other considerations	Certainty	Importancy
11	observational	serious	serious	Not serious	Not serious	None	⨁◯◯◯ Very low	IMPORTANT

Only a subset of the included studies stratified participants according to disease severity or cognitive status, whereas most compared patients with Parkinson's disease and healthy controls only. Consequently, evidence regarding associations with disease severity was limited to a small number of studies.

### 3.2 Methods Used for Microbiota Analysis

Analysis of the methods used in the studies revealed that the majority used 16S rRNA gene sequencing, a widely recognized technique for identifying and characterizing the taxonomic composition of the microbiome. However, there was variation in the hypervariable regions analyzed, reflecting different approaches to increasing taxonomic resolution (Figure 2). The V1‐V3 regions were explored by Pereira et al. [14] and Yay et al. [17, 18], while V3‐V4 was the most frequently used by the other articles [10, 12, 15, 16, 19]. Other studies focused on the V4 [11] and V4‐V5 [13] regions, highlighting the methodological diversity in investigating the oral microbiota. In addition, the most common sequencing platforms were Illumina MiSeq and its variants, demonstrating a trend towards technological standardization among studies, which makes it easier to compare findings.

**FIGURE 2:**
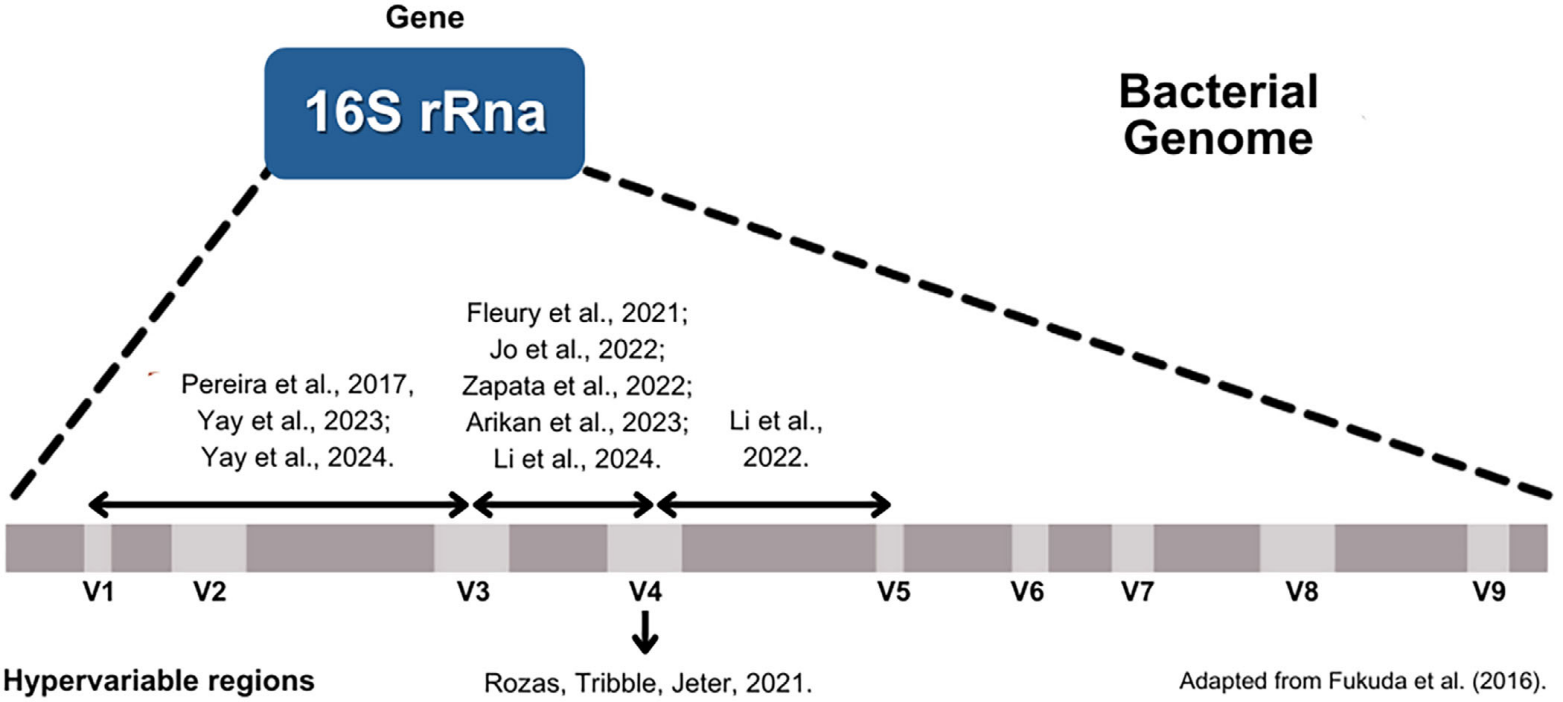
Distribution of hypervariable regions studied by the authors.

Although 16S rRNA gene sequencing was predominant, two studies adopted complementary methodologies, broadening understanding of the microbiome. Arikan et al. [16] included metaproteomic analysis, allowing not only taxonomic identification, but also functional investigation of the microbiome. Mihaila et al. [9] opted to sequence microbial RNA instead of genomic DNA, using the Trizol method followed by mini column purification, to analyze gene expression of the oral microbiota. These additional approaches provide insights into the metabolic activity of microbial communities, complementing findings obtained by conventional sequencing.

### 3.3 Most Frequent Microorganisms in Parkinson's Disease

In this review, the microorganisms identified were organized according to their taxonomic classifications, covering phylum (p_), class (c_), order (o_), family (f_), genus (g_) and species (s_), each represented by a corresponding letter. Not all taxonomic classes were mentioned in all the studies included, but all the microorganisms presented as the most frequent were considered in the data collection, ensuring comprehensive inclusion of the available evidence. This systematization allowed for a more detailed analysis of the oral microbiota associated with PD.

The homogeneity of the data varied between the studies, reflecting the diversity of the methodological approaches and contexts investigated. While some studies analyzed broader taxonomic categories, others focused on more specific levels. The families *Lactobacillaceae* [10, 14] and *Prevotellaceae* [13, 14], as well as the phylum *Firmicutes* [10, 13], were mentioned in only two studies each, while the class *Bacilli* and the order *Lactobacillales* were cited in only one study 13].

Most articles focus on the identification of specific genera and species, allowing a more detailed analysis of the microbial composition. Among the genera identified, *Lactobacillus* stands out as the one most associated with PD, appearing in 45.45% of the references [9, 11, 12, 15, 19]. Next, *Prevotella* [13, 14, 15, 16, 19] and *Streptococcus* [11, 13, 14, 16] appear with a frequency of 36.7% each, indicating that they were among the genera most frequently reported in association with PD. Other genera, such as *Veillonella*, *Megasphaera* and *Scardovia*, were also considered, although less expressively, with an association of 27.3% [9, 10, 12, 14, 16, 19].

Among the most prevalent species were Tannerella forsythia [11, 18], Prevotella nigrescens [11, 17], Porphyromonas gingivalis [15, 19], Parascardovia denticolens, Lactobacillus fermentum and Lactobacillus acidophilus [9, 11], each with a frequency of 18.2%. On the other hand, some species such as Streptococcus pneumoniae and Mycoplasma orale, although they have also been identified in the oral microbiota of Parkinson's patients, were observed in only one study each [11], representing 9.1% of the sample and indicating that their role still needs to be further investigated.

The other bacterial groups associated with PD are shown in Figure 3, providing a clear visualization of the prevalence and distribution of microorganisms in the oral microbiota of patients with the disease. This diversity of findings reinforces the need for methodological standardization and additional studies to better understand the relationship between the oral microbiota and PD.

**FIGURE 3:**
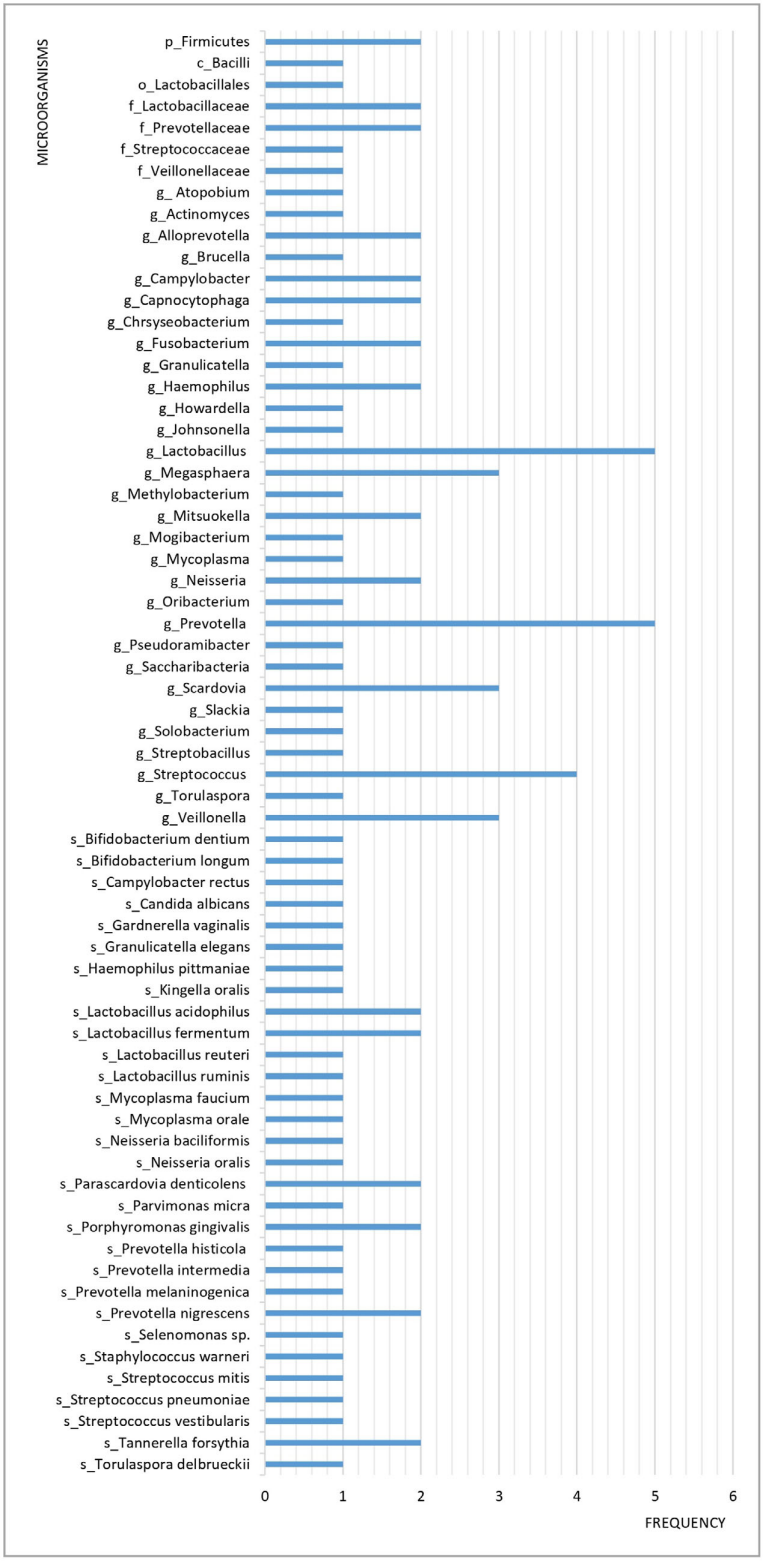
Frequency of the most cited oral microorganisms in Parkinson's disease.

### 3.4 Association of Oral Microorganisms With Disease Severity and Clinical Features

Only four studies evaluated associations between oral microbiota and disease severity or cognitive status. Therefore, the findings presented below are based on this subset of studies. Higher abundance of *Veillonella* was reported across comparisons involving healthy controls, mild PD, and advanced PD, suggesting an association with increasing disease severity [14, 16]. *Neisseria*, on the other hand, showed a drastic reduction in the advanced stages of cognitive impairment [13, 14, 16].

A positive association was observed between the abundance of *Lactobacillus* species and greater disease severity or worse clinical status in the included studies [9, 11, 12, 19]. Specifically, the *Lactobacillus reuteri* species was associated with poorer motor performance, as well as being related to increased alpha‐synuclein secretion [9, 12]. *Lactobacillus acidophilus* has been associated with motor decline [11]. A significant decrease in a single bacteriophage (*Streptococcus phage PhiSpn 200*) and increases in three yeast species (*Candida albicans, Candida dubliniensis* and *Saccharomyces cerevisiae*) have also been observed in individuals with PD [9].

One study investigated microbial diversity in soft and hard tissues separately [11]. Soft tissue samples from PD patients showed a higher abundance of potentially pathogenic oral species from the genera *Lactobacillus*, *Tannerella forsythia* and *Prevotella intermedia*. In hard tissues, *Fusobacterium, Capnocytophaga, Alloprevotella, Prevotella* and *Campylobacter rectus* were more frequent. The abundance of *Streptococcus pneumoniae* was higher in patients with PD and was also associated with the presence of dysphagia [11].

In saliva, a positive correlation was observed between Actinomycetales and tremor scores, and between Veillonella and dysphagia [10].

Some studies indicate that bacterial species present in the oral cavity, such as *Treponema denticola, Prevotella melaninogenica* and *Porphyromonas gingivalis*, are more prevalent in PD patients and may be linked to the degradation of the blood‐brain barrier or the release of toxins that reach the central nervous system [15, 17, 18]. In addition, the reduced diversity of the microbiota in PD patients, associated with an increase in periodontal pathogens, may aggravate dysbiosis and contribute to an increase in systemic inflammatory cytokines [17, 19].

### 3.5 Alpha and Beta Diversity

Alpha and beta diversity metrics are widely used to characterize the structure of microbial communities. While alpha diversity summarizes the richness and uniformity of species within the same sample, beta diversity evaluates the differences in microbial composition between different groups, considering both abundance and the presence or absence of sequences [33].

Analysis of these parameters in the studies analyzed revealed varied results (figure 4). Of the articles included, 64.0% of the studies found no statistically significant differences in the alpha diversity of the samples [9, 11, 12, 13, 14, 16, 19]. One study indicated a lower alpha diversity in patients with PD, especially in oral samples, suggesting a possible reduction in the ecological stability of the oral microbiota and a greater susceptibility to the growth of opportunistic microorganisms [10]. In contrast, in beta diversity, 82.0% of the studies found significant differences between healthy and PD patients, reinforcing the hypothesis of dysbiosis in patients, in which the composition of the oral microbiota of these individuals deviates from the profile observed in healthy individuals [11, 12, 13, 14, 15, 16, 17, 18, 19].

**FIGURE 4:**
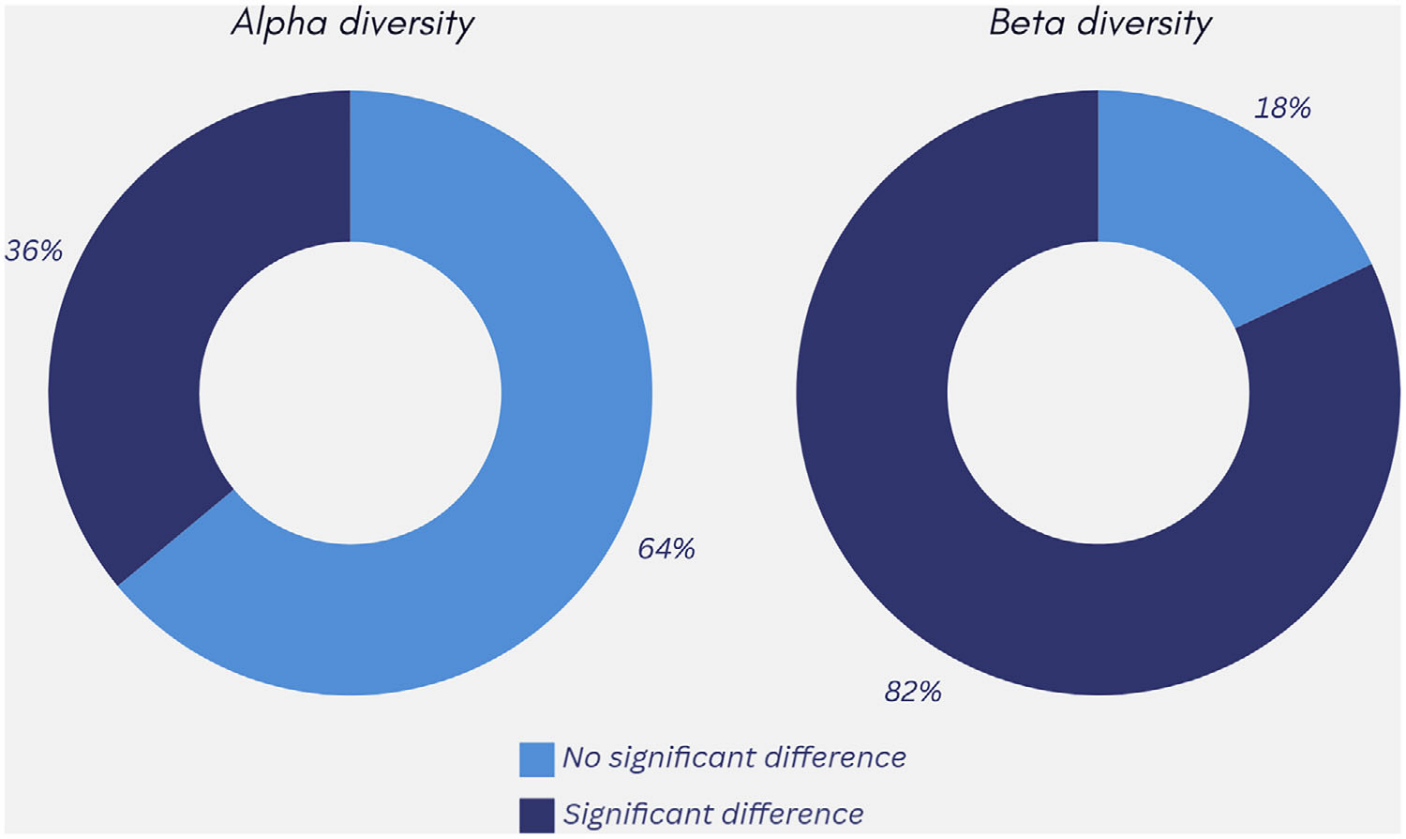
Alpha and beta diversities and their significance in the included studies.

However, not all studies have identified these alterations. Mihaila et al. [9] found no significant differences in alpha or beta diversity indices. One study did not provide information on beta diversity in the samples [10]. This discrepancy may be related to methodological differences between studies, such as variations in participant inclusion criteria, collection methods, and microbiome analysis techniques.

## 4 Discussion

The findings of this systematic review suggest that Parkinson's disease is associated with alterations in oral microbiota composition. Although several microbial taxa were consistently reported across the included studies, the available evidence is derived predominantly from observational studies, which precludes establishing causal relationships or confirming a role of oral dysbiosis in disease progression.

The oral microbiota is considered the second most diverse microbial community in the human body [34], playing an essential role in maintaining oral and systemic health. Its association with neurological diseases has been increasingly investigated, especially in conditions like PD, where oral dysbiosis is believed to influence both motor and non‐motor symptoms.

The studies analyzed in this review highlighted the potential of specific microbial taxa as candidate biomarkers. However, these findings require validation in longitudinal studies before clinical application. For example, Arikan et al. [16] suggested that the oral microbiota is dynamic and that specific microbial changes are associated with clinical characteristics of PD. In particular, a lower relative abundance of *Neisseria* was significantly correlated with poorer cognitive performance. Similar findings were reported by Pereira et al. [14] and Li et al. [13], supporting the hypothesis that alterations in the oral microbiota, particularly involving *Neisseria*, may be associated with cognitive impairment in patients with PD. These findings suggest that specific oral microbial taxa may represent promising candidate biomarkers; however, their clinical utility requires validation in well‐designed longitudinal studies.

Another aspect addressed by the studies was the relationship between oral dysbiosis and the severity of motor symptoms. Rozas, Tribble, and Jeter [11] reported that genera such as *Streptococcus* and *Veillonella* were associated with more severe motor symptoms, while Li et al. [13] pointed out a reduction in the functional capacity of the microbiota, such as amino acid metabolism, in patients with greater motor impairment. Mihaila et al. [9] complemented this view by demonstrating that species such as *Lactobacillus acidophilus* were related to motor decline, in addition to associating inflammatory markers with disease severity.

Non‐motor symptoms, including xerostomia, also showed relevant correlations with the oral microbiota. Evidence in the literature indicates that xerostomic patients exhibit significant changes in the microbial composition of the oral cavity [35, 36], suggesting that oral dysbiosis may be associated with the occurrence and severity of these symptoms.

Fleury et al. [10] highlighted the association between higher prevalence of *Lactobacillus* and worsening of xerostomia, linking this condition to higher levels of oral inflammation. This connection was also explored by Zapata et al. [15], who identified severe dysbiosis related to the progression of non‐motor symptoms. Although *Lactobacillus* is widely used in probiotics, such evidence raises the possibility that, in certain contexts, its presence may be harmful. Thus, alterations in the oral microbiota may contribute to reduced quality of life by being associated with symptoms that extend beyond motor impairment.

Taken together, these findings suggest that changes involving *Neisseria*, *Lactobacillus*, and other microbial taxa deserve further investigation as candidate biomarkers associated with cognitive impairment and disease severity. However, their predictive value cannot be established based on the currently available evidence.

Li et al. [19] presented relevant data on the influence of the oral microbiota on cognition, demonstrating significant differences in the relative abundance of *Prevotella*, *Lactobacillus*, *Megasphaera*, *Atopobium*, and *Howardella* in patients with PD and mild cognitive impairment. These changes were negatively correlated with MMSE and MoCA scores, suggesting an association with poorer cognitive performance. Furthermore, the predictive functional changes identified, such as purine, pyrimidine, and amino acid metabolism, reinforce the hypothesis that the oral microbiota may be associated with metabolic pathways potentially relevant to PD.

We know that the balance of the microbiota is influenced by a variety of endogenous and exogenous factors [37]. However, significant gaps still exist in understanding this relationship. Most current studies focus on identifying microbial patterns at different stages of PD, but few robustly explore the predictive potential of these alterations. Future investigations should include longitudinal studies that monitor patients over time, as well as clinical trials aimed at restoring microbial balance as a therapeutic strategy.

In parallel, Yay et al. [17, 18] reported that patients with PD and periodontitis present a high prevalence of species associated with periodontal dysbiosis, such as *Treponema denticola*, *Prevotella melaninogenica*, and *Parvimonas micra*. These findings, combined with changes in beta diversity and abundance of salivary species, suggest that chronic inflammatory conditions in the oral cavity may be associated with differences in oral microbial composition observed in patients with PD.

However, it is important to note the limitations of this study when investigating the oral microbiota in patients with PD and periodontitis. Periodontitis is known to induce a specific and well‐established dysbiosis [38, 39, 40]. The microbial profile in periodontitis is often dominated by pathogens known to thrive in an inflamed periodontal environment [40], which could obscure the identification of microbial changes directly associated with PD. Therefore, future studies should consider control groups without periodontal disease or explore more refined methods to separate the effects of these two conditions on the oral microbiota.

The relationship between microbial diversity, both alpha and beta, and cognitive decline also deserves special attention, considering that lower diversity may reflect a less resilient microbial environment, more prone to specific dysbioses [41]. Although 64.0% of the studies did not identify significant differences in alpha diversity [9, 11, 12, 13, 14, 16, 19], the identification of reduced diversity in certain patients with PD suggests that this lower ecological resilience may favor the development of dysbiosis.

On the other hand, beta diversity showed more consistent differences between patients with PD and healthy individuals [11, 12, 13, 14, 15, 16, 17, 18, 19], indicating a clear alteration in the oral microbiota composition associated with the disease. These changes in beta diversity reinforce the hypothesis that the oral microbiota of PD patients deviates from a healthy profile, possibly contributing to harmful inflammatory or immune processes.

Dietary factors may also influence oral microbiota composition. One included study reported that patients with PD had lower intake of fiber, fruits, and vegetables and higher consumption of animal proteins and fats. Such dietary patterns may contribute to alterations in oral microbial composition and should be considered in future studies investigating oral microbiota in PD [15].

## 5 Limitations

This review should be interpreted considering the limitations of the currently available evidence. Most included studies used case‐control or cross‐sectional designs, which do not allow temporal relationships or causal inferences between oral microbiota alterations and PD to be established. In addition, considerable methodological heterogeneity was observed regarding sampling sites, sequencing protocols, taxonomic resolution, and reported outcomes, which precluded quantitative synthesis through meta‐analysis. Only a minority of the included studies stratified participants according to disease severity or cognitive status, limiting comparisons across different clinical stages.

Another important consideration is that some included studies evaluated patients with periodontitis. Because periodontal disease is itself associated with well‐established oral dysbiosis, periodontal status may have acted as a confounding factor, making it difficult to distinguish microbial alterations related to PD from those associated with periodontal inflammation. Nevertheless, these studies were retained because of the limited number of available investigations addressing oral microbiota in Parkinson's disease and because they provide relevant information on the coexistence of both conditions.

Despite these limitations, the consistency of several microbial findings across independent studies supports an association between PD and alterations in oral microbiota composition. Future longitudinal studies using standardized microbiome methodologies, with careful characterization of periodontal status and disease severity, are needed to validate these findings and clarify their clinical relevance.

## 6 Conclusion

The available evidence suggests that PD is associated with alterations in oral microbiota composition. Across the included studies, the genera *Lactobacillus*, *Prevotella*, and *Streptococcus*, as well as the species *Tannerella forsythia*, *Prevotella nigrescens*, *Parascardovia denticolens*, *Porphyromonas gingivalis*, *Lactobacillus fermentum*, and *Lactobacillus acidophilus*, were the microorganisms most frequently associated with PD. However, because the available evidence is derived predominantly from observational case‐control and cross‐sectional studies, causal relationships and the role of oral dysbiosis in disease progression cannot be established. These microbial signatures should therefore be considered promising candidate biomarkers that require validation in well‐designed longitudinal studies using standardized taxonomic and functional microbiome analyses before their clinical applicability can be determined.

## Funding

This authors have nothing to report.

## Conflicts of Interest

The authors declare no conflicts of interest.

## Ethical Statement

This article does not contain any studies with human participants or animals performed by any of the authors.

## Data Availability

The data supporting the findings of this study are available within the articles included in the systematic review, which are properly cited in the manuscript. No new primary data were generated. The search strategy, eligibility criteria, and data extraction process can be made available from the corresponding author upon reasonable request.
